# MicroRNAs in Hyperglycemia Induced Endothelial Cell Dysfunction

**DOI:** 10.3390/ijms17040518

**Published:** 2016-04-07

**Authors:** Maskomani Silambarasan, Jun Rong Tan, Dwi Setyowati Karolina, Arunmozhiarasi Armugam, Charanjit Kaur, Kandiah Jeyaseelan

**Affiliations:** 1 Department of Biochemistry, NUS Medicine, National University of Singapore, Singapore 117596, Singapore; m.s19@nus.edu.sg (M.S.); bchtjr@nus.edu.sg (J.R.T.); setyowatikd1@gis.a-star.edu.sg (D.S.K.); bchaa@nus.edu.sg (A.A.); 2 Department of Anatomy, NUS Medicine, National University of Singapore, Singapore 117594, Singapore; charanjit_kaur@nuhs.edu.sg; 3 Department of Anatomy and Developmental Biology, School of Biomedical Sciences, Faculty of Medicine, Nursing and Health Sciences, Monash University, Melbourne, Clayton VIC 3800, Australia

**Keywords:** microRNA, diabetes mellitus, endothelial dysfunction, apoptosis, hyperglycemia

## Abstract

Hyperglycemia is closely associated with prediabetes and Type 2 Diabetes Mellitus. Hyperglycemia increases the risk of vascular complications such as diabetic retinopathy, diabetic nephropathy, peripheral vascular disease and cerebro/cardiovascular diseases. Under hyperglycemic conditions, the endothelial cells become dysfunctional. In this study, we investigated the miRNA expression changes in human umbilical vein endothelial cells exposed to different glucose concentrations (5, 10, 25 and 40 mM glucose) and at various time intervals (6, 12, 24 and 48 h). miRNA microarray analyses showed that there is a correlation between hyperglycemia induced endothelial dysfunction and miRNA expression. *In silico* pathways analyses on the altered miRNA expression showed that the majority of the affected biological pathways appeared to be associated to endothelial cell dysfunction and apoptosis. We found the expression of ten miRNAs (miR-26a-5p, -26b-5p, 29b-3p, -29c-3p, -125b-1-3p, -130b-3p, -140-5p, -192-5p, -221-3p and -320a) to increase gradually with increasing concentration of glucose. These miRNAs were also found to be involved in endothelial dysfunction. At least seven of them, miR-29b-3p, -29c-3p, -125b-1-3p, -130b-3p, -221-3p, -320a and -192-5p, can be correlated to endothelial cell apoptosis.

## 1. Introduction

Physiologically, hyperglycemia is often caused by metabolic abnormalities. The early metabolic abnormalities are termed the impaired fasting glucose (IFG), a pre-diabetic stage. It may take several years before the IFG eventually develop Type 2 Diabetes Mellitus (T2DM) [1]. Hyperglycemia induces both phenotypic and genotypic alterations in vascular tissues. The effects of hyperglycemia are often irreversible and lead to progressive cell dysfunction. Chronic exposure to hyperglycemia is identified as the primary casual factor in the pathogenesis of diabetic complications and in the development of endothelial dysfunction [2]. Evidently, hyperglycemia is considered as an important risk factor for cardiovascular diseases. Therefore, early detection and aggressive treatment of hyperglycemia will prove to be useful in retarding the development of microvascular complications, as well as prevention of macrovascular complications [3].

Endothelial cells (ECs) are simple squamous cells that line the luminal surface of blood vessels, which serve as an interface between circulating blood and the intima layer of the blood vessels [4,5]. ECs are crucial for the maintenance of healthy vasculature and are sensitive to changes to blood glucose level [6]. Under normal condition, ECs remain in the quiescent state and regulate vascular tone. While during T2DM disease progression, hyperglycemic conditions cause functional impairment of ECs resulting in endothelial dysfunction [7]. This event could be characterized by decreased nitric oxide production, enhanced endothelial permeability, elevated adhesion molecule expression and increased cell death [8]. These ultimately lead to T2DM-related vascular complications [9]. Hence, detection and prevention of vascular damage in the early stages of T2DM progression can ameliorate the onset of vascular complications.

The current biomarkers for endothelial dysfunction such as C-reactive protein (CRP), P-selectin, E-selectin, Von Willebrand factor (VWF), Interleukin 6 (IL-6), Chemokine C-C motif ligand 2 (CCL2), Vascular cell adhesion molecule 1 (VCAM-1), Intercellular adhesion molecule 1 (ICAM-1) can only be used in later stage of T2DM disease progression [10]. Most of the current techniques in monitoring glucose level cannot quantify the degree of endothelial cell damage [6,11]. There is a need to identify marker(s) that can be sensitive enough to detect endothelial dysfunction in blood early on, and growing evidence in the literature supports that microRNAs can potentially function as such marker(s) [12,13,14].

MicroRNAs (miRNAs) are a class of small (20–24 nucleotides), endogenously expressed, non-coding RNA molecules [15]. They play pivotal roles in the regulation of gene expression that controls a wide range of biological functions such as cellular metabolism, proliferation, differentiation and apoptosis [16]. The altered expression and role of miRNAs in vascular and metabolic perturbation is widely studied [17]. Zampetaki *et al.* [18] have demonstrated that circulating miRNA is altered in diabetic patients and that expression of a panel of miRNAs could predict the development of diabetes in otherwise normal individual. We have also shown that circulating blood miRNAs are dysregulated in T2DM [19].

Our hypothesis is that, exposure of vascular endothelial cells to hyperglycemic conditions will lead to endothelial cell dysfunction and it could manifest in the altered expression of their miRNA profiles. Thus, the aim of this study is to: (1) identify miRNA that are involved in endothelial dysfunction in hyperglycemic state that could lead to the elucidation of the glucose responsive miRNAs which could prove useful for identification of hyperglycemia induced vascular complications; (2) determine if these miRNAs could be potentially used as biomarker which could well differentiate the impaired fasting glucose (IFG) from T2DM.

We reanalyzed our previous findings on dysregulations of miRNA and mRNA expression in both diabetes and pre-diabetes (IFG) stages [19,20]. These human blood miRNA profiles were then compared to an *in vivo* diabetes (rat) model. The miRNA that were altered in both the human IFG/T2DM and rat T2DM were then compared to the *in vitro* laboratory model of human umbilical vein endothelial cells (HUVEC) exposed to hyperglycemic conditions. HUVEC cells have been used widely in vascular endothelial cell based research [21,22,23,24], and it has been proposed to be an ideal candidate for such studies. The genes and miRNAs expression profiling of various endothelial cells showed that most of them are clustering closely with HUVECs [25,26].

Interestingly, even though the three experiments are entirely independent/different from each other, with one common factor, namely the hyperglycemic condition, they could identify common miRNAs that are significantly altered in among them. The expression of ten miRNAs, miR-26a-5p, -26b-5p, 29b-3p, -29c-3p, -125b-1-3p, -130b-3p, -140-5p, -192-5p, -221-3p and -320a were observed to be gradually increased with increase in glucose concentration. It is noteworthy that, among these, seven miRNAs (miR-29b-3p, -29c-3p, -125b-1-3p, -130b-3p, -221-3p, -320a and -192-5p) have been reported to be associated with endothelial cell apoptosis.

## 2. Results

### 2.1. Glucose Uptake Measurement Assay

As our aim was to identify the impact of high glucose concentration on miRNA expression profiles on the vascular endothelium, we selected HUVEC system to carry out our experiments.

**Figure 1:**
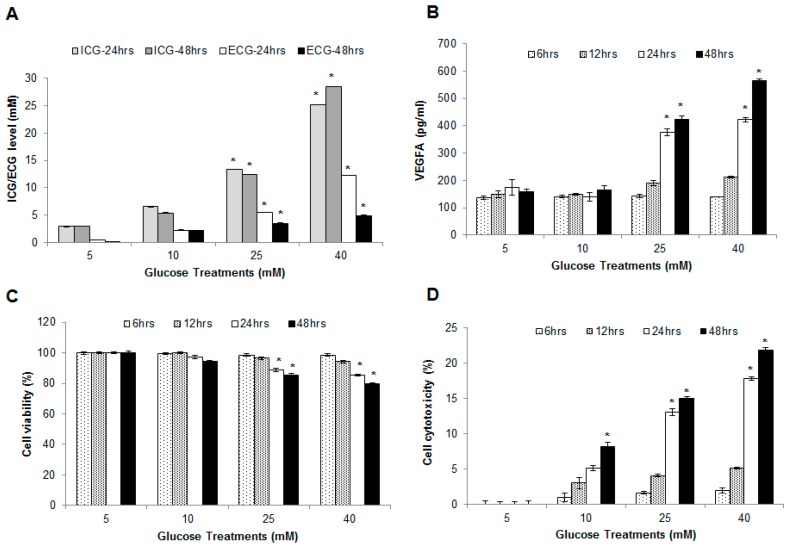
HUVECs subjected to various glucose treatments: (**A**) glucose assay; (**B**) vascular endothelial growth factor A (VEGFA) concentration in culture media; (**C**) cell viability assay; and (**D**) cytotoxicity (LDH) assay. ICG, Intracellular Glucose; ECG, Extracellular Glucose. Cells were treated with various glucose concentrations (5, 10, 25 and 40 mM). Cells and media were collected at 6, 12, 24 and 48 h. Each experiment was carried out in triplicates and as sets of three independent experiments (*n* = 3). Data presented as mean ± SEM; * Indicates statistical significance *p* < 0.05 using student *t*-test against control (test *vs.* 5 mM Glucose treatment).

In order to check whether the glucose concentrations within the cells do vary in accordance with the changes in the external concentrations of glucose, we determined the intracellular level of glucose corresponding to each treatment (5 to 40 mM). We observed an increasing level of glucose within the cells at 2.90, 6.55, 13.37 and 25.20 mM when the HUVEC cells were exposed to 5, 10, 25 and 40 mM glucose (in media), respectively (Figure 1A). We have also measured the residual glucose concentration in the culture media. From the results, we could observe a fraction of the total glucose in the medium, as expected, has been metabolized as well.

### 2.2. Hyperglycemia Induced Endothelial Dysfunction

Vascular Endothelial Growth Factor (VEGFA) in the culture media was measured to determine if high glucose (25 and 40 mM) concentration induces secretion of VEGFA from the endothelial cells. The data (Figure 1B) showed that there was a significant increase in VEGFA upon treatment with higher concentrations of glucose for 24 and 48 h. Both 5 and 10 mM glucose treatments did not show significant changes in VEGFA level.

### 2.3. Effect of Hyperglycemia on Cell Viability and Cytotoxicity in HUVECs

Cell viability and cytotoxicity were assessed using 3-[4,5-dimethylthiazol-2-yl]-2,5-diphenyl tetrazolium bromide (MTT) and Lactate dehydrogenase (LDH) assay, respectively. The data from MTT assay showed that as glucose level increased from that of control (5 mM), the cell viability decreased by 0.6%, 11.37% and 14.8% at 10, 25 and 40 mM glucose after 24 h of incubation, respectively. Further decrease in cell viability (2.9%, 14.5%, 21.4% for 10, 25 and 40 mM glucose treatments, respectively) was observed when incubation time was prolonged up to 48 h (Figure 1C). An increase in LDH activity in a concentration and time dependent manner (Figure 1D) was also observed. The cell cytotoxicity results inversely correlated to the cell viability data.

### 2.4. Glucose Induced Endothelial Apoptosis

**Figure 2:**
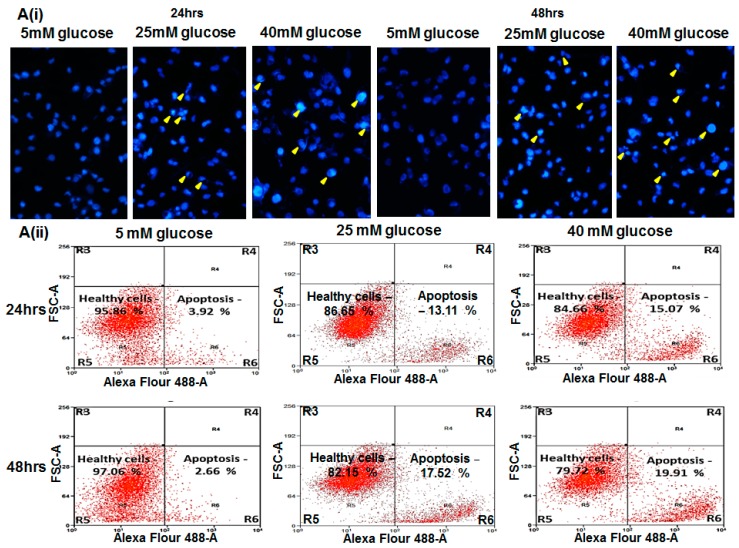

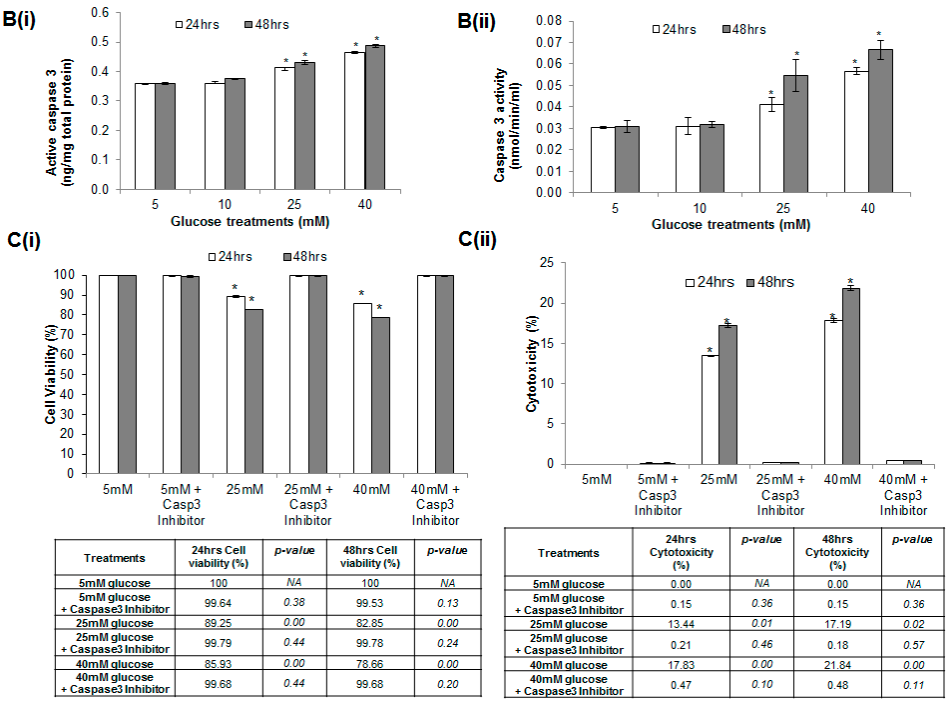
Endothelial cell apoptosis: (**A**) Representative data from 5, 25 and 40 mM glucose treatment at 24 and 48 h, (**i**) DAPI nuclear staining: The images were captured using Olympus IX51 microscope (20× magnification). Healthy nuclei can be observed for 5 mM glucose treatments (24 and 48 h). Condensed and pyknotic nuclei can be seen under hyperglycemic conditions at both 25 and 40 mM glucose treatment for 24 and 48 h, respectively (yellow arrowhead); (**ii**) Dot-plot graph on fluorescence-activated cell sorting (FACS) Annexin V staining. The percentage of healthy cells and apoptotic cells can be seen in R5 and R6 quadrants, respectively. Figure S3 (Supplementary Material) shows the histogram and statistical analysis computed for the DAPI stained unhealthy cells, Annexin V positive cells and Annexin V and Ethidium Homodimer III stained cells; (**B**) (**i**) Active Caspase 3 and (**ii**) Caspase 3 activity were measured by colorimetric readings and absorption of fluorescence released from the cleaved substrate (DEVD-AMC), respectively; (**C**) Cell viability (**i**) and cytotoxicity (**ii**) assay for HUVEC cells exposed to hyperglycemia in the presence of caspase 3 inhibitor. Table shows the significance *t*-test *p*-values for each treatment. All data presented as mean ± SEM (*n* = 3); * Indicates statistical significance *p* < 0.05 using *t*-test (test *vs.* 5 mM Glucose treatment).

Both the MTT and LDH assays above showed that the EC cell viability is decreased in the presence of high glucose. We also observed that the phenotype of the cells changes with hyperglycemic condition. The nuclei of the HUVEC cells in hyperglycemic media were observed to be either condensed or pyknotic especially at 40 mM glucose at 24 and 48 h treatments (Figure 2Ai). However, we found that these changes (in nuclear morphology) were not statistically significant (*p* > 0.05; Figure S1, Supplementary Material) for short term (6 and 12 h) glucose treatments. The association between hyperglycemia induced decrease in EC viability and endothelial apoptosis have been documented [24,27].

We carried out 3-dye staining (DAPI, Annexin V and Ethidium Homodimer III) of the cells followed by flow cytometry assay to further confirm that HUVECs undergo apoptosis during hyperglycemic treatments. We could observe an increase in the percentage of cells with shrunken nuclei (2.29%, 10.37% and 13.24% at 10, 25 and 40 mM glucose, respectively) after 24 h of incubation (Figure S2A (Supplementary Material), 24 h panel). The percentage of cells with shrunken nuclei increased further (9.42%, 12.34%, 15.34% at 10, 25 and 40 mM glucose, respectively) after 48 h of incubation (Figure S2A, 48 h panel). Furthermore, during apoptosis, the exposed phosphatidylserine residues on the cell surface can be labeled with Annexin V. Hence, we plotted the flow cytometry raw data for FCS-A *vs.* Alexa Fluor 488A (Annexin V staining) which specifically indicated apoptotic cells only. The results showed an increase in apoptotic cells in R6 quadrant with higher concentration of glucose treatment at 24 and 48 h, respectively (Figure 2Aii and Figure S2B).

In order to differentiate necrosis as well as early and late apoptosis, we have also examined the cells stained for PE-Texas Red-A (Ethidium Homodimer III stained cells) against cells stained for Alexa Fluor 488A (Annexin V stained cells). Figure S2C shows that the cells stained with Annexin V remained only in R13 quadrant indicating that the cells were in the stage of early apoptosis. Quadrant R11 shows cells that were stained with both Annexin V and Ethidium Homodimer III and hence they represented the cells in late apoptosis. From these results, it is also clear that the HUVEC cells undergo apoptosis in a glucose concentration and time dependent manner.

### 2.5. Hyperglycemia Induced Caspase-Mediated Apoptosis in HUVECs

Having examined endothelial cell apoptosis upon hyperglycemia, we wanted to understand the underlying molecular events leading to it. For this, we carried out the measurement of both active caspase-3 protein level and caspase-3 activity in the total cell lysates at different concentrations of glucose for both 24 and 48 h. Compared to the control (5 mM glucose grown cells), treatment at 25 and 40 mM glucose showed a significant increase in the active form of caspase-3 protein that correlated with its activity (using DEVD-AMC as substrate). Moreover, both the caspase-3 protein and activity level increased further at 48 h time exposure (Figure 2Bi and Figure 2Bii). Concomitantly, HUVEC cells that were exposed to 5, 25 and 40 mM glucose (24 and 48 h) and treated with 25 µM caspase 3 inhibitor (Ac-DEVD-CHO; Alexis Biochemicals, Farmingdale, NY, USA) showed that cell viability has improved and cell cytotoxicity has been alleviated (Figure 2Ci and Figure 2Cii).

Hence, from these results, we could interpret that hyperglycemia induced endothelial cell (HUVEC) apoptosis in our study is possibly mediated through a caspase-3 dependent pathway.

### 2.6. miRNAs Correlating with Increase in Hyperglycemia

**Figure 3:**
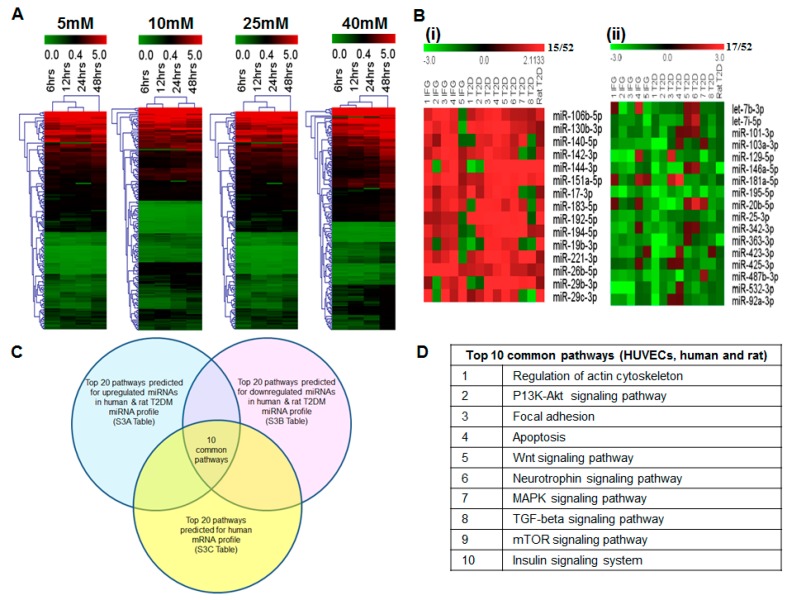
miRNA microarray and pathway analysis. (**A**) Hierarchical clustering of miRNAs in HUVECs subjected to different glucose treatments (5, 10, 25, and 40 mM) at 6, 12, 24 and 48 h. In total, 177 miRNAs with background subtracted mean signal intensities ≥300 are included; (**B**) Heat map of selected 52 miRNAs dysregulated in human and rat. miRNAs that showed differential expression are grouped into two categories (**i**,**ii**) of miRNAs that remained (**i**) upregulated in both impaired fasting glucose (IFG) and T2DM (**ii**) downregulated in both IFG and T2DM *vs.* controls. Data are expressed as fold change. Red represents up-regulation; green indicates down-regulation and grey indicates not detected; (**C**) Venn diagram showing results of pathway analysis for dysregulated miRNAs and mRNAs using DIANA miRPath 2.0 and WebGestalt, respectively; (**D**) Ten common pathways that could possibly participate in endothelial dysfunction are also shown.

miRNAs showing dysregulation during pathophysiological conditions have been linked to the gene and consequently protein expression in cells. Thus, we postulated that miRNAs responding to changes in glucose concentrations could possibly be involved in pathways leading to apoptosis and endothelial dysfunction in this study. Thus, we performed miRNA microarray on HUVECs treated with various glucose concentrations (5 to 40 mM) and at various time-points (6 to 48 h). Our microarray data have been deposited at the NCBI database under the GEO Accession No: GSE74296, GPL21059. Among these miRNAs, we were able to identify 177 miRNAs (with a signal intensity >300) that were commonly dysregulated in all time points. The hierarchical clustering of these miRNAs showed that miRNA expression pattern changes in all glucose treatments (Figure 3A). Among them, a total of 62 miRNAs showed a gradual increase from 6 to 48 h for 5, 10, 25 and 40 mM glucose (Table S1, Supplementary Material).

### 2.7. Blood miRNA Expression Profiles in Individuals with IFG and T2DM

We were interested in identifying the miRNAs that are responsive to glucose concentration and could be used as indicators of endothelial dysfunction, especially in a pathophysiological condition such as in the pre-diabetes (IFG) and diabetes (T2DM). We re-analyzed the human IFG/T2DM blood miRNA expression profile data (GEO Accession No: GSE26167; and SuperSeries GSE26168) [20]. It is noteworthy that the individuals/subjects that were recruited to the study [20] do not have any existing clinical complications or on any medications. They were the newly diagnosed IFG or T2DM individuals. miRNAs showing raw signal intensity values ≥300 and tested to be statistically significant (*p* < 0.05) were selected for our analysis. One hundred and seventy seven (177) miRNAs have been found to be differentially expressed among the blood samples of IFG and T2DM compared to control group. These miRNAs were grouped into four categories based on their expression pattern to identify those that are differentially expressed among IFG and T2DM individuals (Figure S4, Supplementary Material). Category 1—miRNAs that were upregulated in both IFG and T2DM patients (Figure S4A); Category 2—miRNAs that were downregulated in both IFG and T2DM (Figure S4B); Category 3—miRNAs that remained downregulated in IFG and upregulated in T2DM (Figure S4C); and Category 4—miRNAs that were upregulated in IFG but downregulated in T2DM (Figure S4D).

### 2.8. Comparison of Blood miRNA Profiles in Type 2 Diabetes Mellitus between Human and Rat

As the patient sample size reported by Karolina *et al.* [20] was relatively small, we also included the blood miRNA profiles of rat T2DM model data published from our group (GEO Accession No: GSE26167; SuperSeries: GSE26168) [20]. Consequently, we compared the expression of the 177 human miRNAs from the four different categories with the rat T2DM blood miRNA profiles. We found a total of 52 miRNAs (Figure 3B) that showed similar expression pattern between the human and rat miRNA profiles during T2DM disease progression. Among these miRNAs, we could observe miR-142-3p to show an increase in the expression both IFG and T2DM (Figure 3Bi). This result also correlated with the three independent diabetes studies carried out by Collares *et al.* [28], Ortega *et al.* [29] and Zhu *et al.* [30]. Most importantly, we could identify the up-regulation of miR-142-3p in IFG condition itself. Hence, miR-142-3p could be considered as an early indicator of hyperglycemia. Moreover, Lalwani *et al.* [31] explored the role of miR-142-3p in relation to vascular dysfunction and reported that overexpression of this miRNA resulted in a loss of vascular integrity. In addition to these, we could also observe an upregulation of miRNAs such as miR-29b-3p, -29c-3p, -144-3p, -183-5p and miR-221-3p as reported previously by others [20,32,33,34,35]. Compared to miR-142-3p, miR-103a-5p was found to be downregulated (Figure 3Bii) in both IFG and T2DM. This observation correlated with the report from Upadhyay *et al.* [36]. They reported that high glucose downregulates miR-103a-5p level in endothelial cells. Xu *et al.* [37] suggested a possible mechanism for miR-103a-5p involving regulation of reactive oxygen species (ROS) during oxidative stress.

We carried out a miRNA microarray for HUVEC exposed to 5, 10, 25 and 40 mM glucose at different time points (6, 12, 24 and 48 h). We found 62 miRNAs that showed a gradual increase from 6 to 48 h for different concentration of glucose. When we compared these 62 miRNAs from our cellular studies analysis with the 52 commonly dysregulated microRNAs from IFG and T2DM of human and rat, we observed 10 miRNAs (miR-26a-5p, -26b-5p, -29b-3p, -29c-3p, -125b-1-3p, -130b-3p, -140-5p, -192-5p, -221-3p and -320a) that exhibited a gradual increase in expression with the increasing glucose concentrations. The increase in expression for the miRNAs was higher in the later time points of 24 and 48 h in cellular studies and it correlated to the expression of these miRNAs in T2DM (Table 1 and Table S2 [38,39,40,41,42,43,44,45,46,47,48,49,50,51,52,53,54,55,56,57,58,59,60,61,62,63,64,65,66,67,68,69,70,71,72,73,74,75,76,77,78,79,80,81,82,83,84,85,86,87]).

### 2.9. In Silico Analysis of miRNA and mRNA Pathways

To understand the role of differentially expressed miRNAs, we performed a biological pathway analysis using DIANA miRpath 2.0 [88] and miRWalk version 2.0 [89]. The 52 miRNAs dysregulated (Figure 3Bi and Figure 3Bii) were used to perform KEGG pathway analysis [90]. The selection criteria used were microT threshold value of 0.8 and cutoff of *p* < 0.05. The results showed more than 100 significantly (*p* < 0.05) dysregulated pathways. Among them, the top 20 pathways were shortlisted (Figure 3C and Table S3).

### 2.10. Analysis of mRNA Profiles of IFG and T2DM (R4)

To gain further insight on endothelial dysfunction during T2DM, we carried out a systematic approach of pathway enrichment analysis using WEB-based Gene SeT AnaLysis Toolkit (WebGestalt) algorithm [91] with the hypergeometric statistical method and multiple corrections using Benjamini-Hochberg (*p* < 0.001). We analyzed our previously published human mRNA microarray data (IFG and T2DM), GEO Accession No: GSE21321 [20]. The top 20 pathways (Table S3) included insulin signaling, VEGF signaling, focal adhesion, regulation of actin cytoskeleton, adherens and tight junction and apoptosis pathways that are associated with T2DM. Most of these pathways are also known to be related to both micro and macrovascular complications during type 2 diabetes [92,93]. From the analysis, we found various endothelial cell specific genes such as nitric oxide synthase (NOS3) and endothelin (EDN1) as well as other genes related to vascular dysfunction such as endothelial permeability (ERG, RHOA, ROCK) [94,95,96,97,98], inflammation (CCL5, TLR4, NFKB1 and IL1B) [99,100,101] and apoptosis (BNIP3, DNM1L, BCL-2, MCL-1 and CASP3) [102,103,104,105,106] to be dysregulated (Table S4). These data clearly indicate that the hyperglycemia can result in endothelial dysfunction and the event can be analyzed from the mRNA data of the blood.

Based on this mRNAs and miRNAs pathways analysis, we were able to deduce 10 common pathways that could possibly participate in endothelial dysfunction (Figure 3C,D).

### 2.11. MicroRNAs as Possible Glucose Responsive and Endothelial Dysfunction Indicators

**Figure 4:**
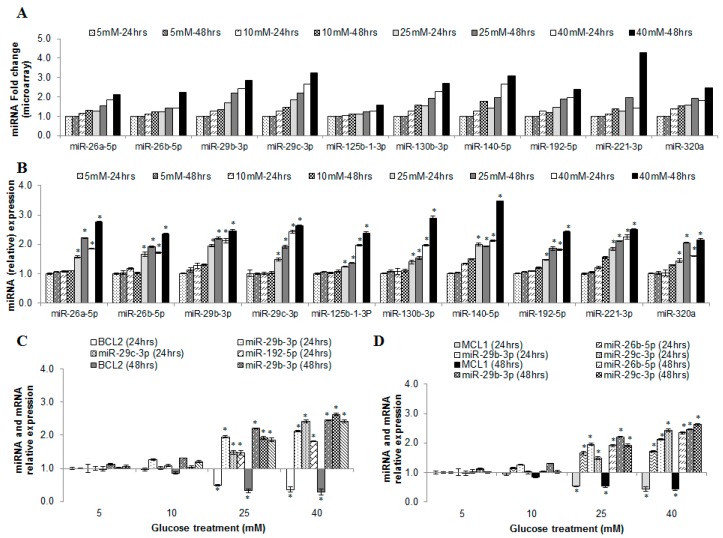
Validation of selected miRNAs and target mRNAs using qPCR. (**A**) Represents the pattern of expression observed for the 10 selected miRNAs from the microarray data for HUVECs treated with different glucose treatments for 24 and 48 h; (**B**) Verification of microarray data in (**A**) by real-time quantitative PCR; (**C**,**D**) The expression level of three miRNAs and their corresponding mRNAs that are involved in apoptosis: (**C**) miR-29b-2p and -192-5p against *BCL2* mRNA and (**D**) miR-26b-5p and -29b-3p against *MCL-1* mRNA. Data are presented as mean ± SEM (*n* = 3). * indicates statistical significance at *p* < 0.05 using t-test against control (test *vs.* 5 mM glucose treatment).

From our microarray data on the HUVEC exposed to hyperglycemia, we selected 10 miRNAs (miR-26a-5p, -26b-5p, -29b-3p, -29c-3p, -125b-1-3p, -130b-3p, -140-5p, -192-5p, -221-3p and -320a) that showed corresponding increase in expression with increase glucose concentration (Figure 4A). These miRNAs were quantitated by stem loop PCR. The results confirmed that all 10 miRNAs were significantly upregulated at higher concentrations of glucose treatments at 24 and 48 h (Figure 4B).

Among these 10, seven microRNAs (miR-26b-5p, -29b-3p, -29c-3p, -130b-3p, -140-5p, -192-5p, and -221-3p) consistently showed an increased expression in both IFG and T2DM (Figure S4A and Table 1). The other three miRNAs (miR-26a-5p, -125b-1-3p and -320a) were found to be downregulated in IFG individuals and subsequently upregulated in T2DM (Figure S4C and Table 1). Notably, these miRNAs remained upregulated in most of our *in vitro* (HUVEC cell culture) study (Table S2, Supplementary Material [38,39,40,41,42,43,44,45,46,47,48,49,50,51,52,53,54,55,56,57,58,59,60,61,62,63,64,65,66,67,68,69,70,71,72,73,74,75,76,77,78,79,80,81,82,83,84,85,86,87]). We have also noticed that *BCL2* and *MCL1* genes that are known to be crucial for the caspase 3 mediated apoptosis can be targeted by miR-26b-5p, -29b-3p and -192-5p. qPCR analysis on both the *BCL2* and *MCL1* genes showed that they are downregulated in our study (Figure 4C,D; Table S2 [38,39,40,41,42,43,44,45,46,47,48,49,50,51,52,53,54,55,56,57,58,59,60,61,62,63,64,65,66,67,68,69,70,71,72,73,74,75,76,77,78,79,80,81,82,83,84,85,86,87]) and an inverse correlation to miR-26b-5p, -29b-3p and -192-5p expression.

## 3. Discussion

Hyperglycemia has been the hallmark and a major risk factor for endothelial dysfunction and vascular complications in diabetes [107]. Under normal physiological conditions, the blood glucose level is tightly regulated in order to maintain the vascular quiescence and integrity. However, in hyperglycemic conditions (uncontrolled glucose level above physiological concentrations, such as in T2DM), endothelial cells (ECs) become dysfunctional and undergo apoptosis [108]. Endothelial dysfunction could be characterized by the decrease in cell viability and an increase in the release of lactate dehydrogenase as well as overproduction of VEGFA [109]. In this study, we have shown that HUVECs exposed to 25 and 40 mM glucose for 24 and 48 h exhibited reduced cell viability followed by an increase in VEGFA secretion. Besides, these cells also showed increased cell apoptosis as indicated by enhanced Annexin V stained cells that corresponded to the increase in glucose concentration (25 and 40 mM) and time (24 and 48 h) as reported [24,27]. We observed that the apoptotic cell death mediated by hyperglycemia is also caspase 3 dependent.

Vascular complications due to T2DM have become one of the most challenging pathology that brings about increased risk for various diseases/complications to individuals [110]. The currently available biomarkers, drugs, and treatments for vascular complications due to T2DM are ineffective in the diagnosis and treatment of the disease, suggesting that our understanding of this metabolic disorder is still incomplete [111]. miRNAs are one of the most fascinating small RNA molecules that regulate gene expression in both normal and pathophysiological conditions. Emerging evidence suggests that miRNAs could fulfill this inadequacy as early biomarkers as well as therapeutic targets or agents [112]. In order to identify the possible role played by miRNAs in the glucose induced endothelial dysfunction/cell death, miRNA microarray data from the *in vitro* hyperglycemic studies, *in vivo* rat model of T2DM and the blood samples of T2DM and IFG individuals were analyzed. Initial analysis from the *in vitro* study (HUVECs exposed to glucose) showed that 177 miRNAs were significantly dysregulated in hyperglycemic conditions with 62 miRNAs (Table S1) showing gradual increase in expression with the increase in glucose concentration. Independent analysis of the miRNA microarray data from the *in vivo* study on T2DM/IFG or T2DM animal model, revealed that a total of 52 miRNAs to be altered in the progression/evolution of T2DM and it correlated to the mRNA expression for the same samples. An unbiased biological pathway analysis was carried out on these two sets of miRNAs independently. Both the biological pathway analyses showed that biological process related to endothelial integrity and function, micro and macrovascular complications and endothelial dysfunction are obviously altered during hyperglycemia.

A more detailed analysis on the miRNA microarray data from the HUVECs exposed to hyperglycemia (*in vitro*) showed that a group of 10 miRNAs (miR-26a-5p, -26b-5p, -29b-3p, -29c-3p, -125b-1-3p, -130b-3p, -140-5p, -192-5p, -221-3p and -320a), gradually increased with increasing glucose concentration at 24 and 48 h treatments. Among them, miR-26a-5p, miR-140-5p, miR-221-3p, miR-29b-3p, miR-192-5p have been implicated in apoptosis (Table S2; [38,39,40,41,42,43,44,45,46,47,48,49,50,51,52,53,54,55,56,57,58,59,60,61,62,63,64,65,66,67,68,69,70,71,72,73,74,75,76,77,78,79,80,81,82,83,84,85,86,87]).

Consistent with our findings, exposure to high glucose has been demonstrated to induce miR-26a-5p expression [113]. Furthermore, Chen *et al.* [114] found that miR-26a-5p increases glucose uptake and suggested that there is a positive feedback loop between increased extracellular glucose concentration and miR-26a-5p expression. Up-regulation of miR-26a-5p have been reported to increase cardiomyocyte apoptosis by increasing reactive oxygen species (ROS) production [115], which is one of the important mediator/pathways for glucose-induced endothelial apoptosis. Apart from these studies, Lezina *et al.* [116] and Zhang *et al.* [117] independently reported that p53 pathway regulates miR-26a-5p expression and induces apoptosis in cancer. We observed that exposure of HUVECs with increasing concentrations of glucose increased glucose uptake as well as miR-26a-5p expression, thus indicating that miR-26a-5p may be considered as a potential glucose responsive miRNAs and a surrogate biomarker for endothelial cell apoptosis.

We found that the expression of miR-140-5p to be increased in IFG as well as in T2DM, in both the human and rat miRNA microarray analyses, as well as in our *in vitro* study, where miR-140-5p level increased with increasing glucose concentrations (Table 1 and Table S2 [72,87,88,89,90,91,92,93,94,95,96,97,98,99,100,101,102,103,104,105,106,107,108,109,110,111,112,113,114,115,116,117,118,119,120,121,122,123,124,125,126,127,128,129,130,131,132,133,134,135]). This increase was also found to correlate with the increase in apoptosis (Figure 2). Lan *et al.* [118] have also reported that treating ovarian cancer cells with miR-140-5p resulted in enhanced apoptosis. Besides, we also observed that the endothelial enriched miR-221-3p remained up-regulated during exposure to hyperglycemia in both *in vitro* and *in vivo* studies, consistent with the findings of Li *et al.* [119], Qin *et al.* [120] and Cerda *et al.* [121]. The authors have reported that up-regulated miR-221 in T2DM could be involved in glucose-induced endothelial apoptosis.

miR-29b-3p was observed to be upregulated in both our *in vitro* and *in vivo* studies. Mott *et al.* [38] reported that miR-29b-3p is involved in endothelial cell apoptosis by inhibiting the anti-apoptotic gene *Mcl-1*. Recently, Ye *et al.* [122] showed that p53 pathway could directly up-regulate miR-192-5p expression and inhibit X-linked inhibitor of apoptosis protein (*XIAP*) which is also an anti-apoptotic gene. Jin *et al.* [123], Geng *et al.* [124] and Cao *et al.* [125] independently showed that miR-192-5p to be involved in the regulation of apoptosis via down-regulation of its target gene *BCL2*. Jin *et al.* [126], also reported that upregulation of miR-192-5p induces apoptosis via suppression of PI3K-Akt signaling pathway. It is noteworthy, that we found PI3K-Akt signaling pathway as one of the top 10 pathways being dysregulated in our analyses. Hence, glucose-induced endothelial apoptosis may be regulated by modulating miR-29b-3p and miR-192-5p.

An independent pathway analysis of the differentially expressed 52 miRNAs (common among human and rat T2DM) revealed that pathways related to vascular dysfunction are affected during T2DM. We also carried out the KEGG pathway analysis for the selected 10 miRNAs that varied with different glucose concentrations. The compilation of the top 10 pathways dysregulated in human, rat and HUVECs studies showed that majority of them are involved in endothelial dysfunction related pathways such as PI3K-Akt signaling, apoptosis, regulation of actin cytoskeleton, focal adhesion, neurotrophin and MAPK signaling. Apoptosis, focal adhesion, adherens and tight junction pathways are dysregulated during T2DM and these pathways could be regulated by the miRNAs [104,105,127]. Hence, the 10 selected miRNAs may potentially help in understanding the mechanisms underlying glucose-induced endothelial dysfunction.

We could observe an opposite profile between the expression of the miRNAs and their corresponding target genes (mRNAs) suggesting that these miRNAs can function as potential indicators of endothelial dysfunction associated apoptosis. miR-26a-5p, -130b-5p, -140-5p, and -221-3p exhibited a positive correlation to the endogenous glucose levels in both the *in vivo* (human and rat samples) and *in vitro* (HUVECs subjected to hyperglycemia) studies (Table 1 and Table S2 [38,39,40,41,42,43,44,45,46,47,48,49,50,51,52,53,54,55,56,57,58,59,60,61,62,63,64,65,66,67,68,69,70,71,72,73,74,75,76,77,78,79,80,81,82,83,84,85,86,87]). Thus, these four miRNAs could be potentially useful as “glucose responsive miRNAs” to detect or identify hyperglycemia or high glucose conditions. We propose that the other three miRNAs (miR-130b-3p, -140-5p and -221-3p) could be triggering endothelial dysfunction via inflammation, pathological angiogenesis, hyperpermeability, apoptosis, and senescence since they have been validated to target several genes (Table S2 [38,39,40,41,42,43,44,45,46,47,48,49,50,51,52,53,54,55,56,57,58,59,60,61,62,63,64,65,66,67,68,69,70,71,72,73,74,75,76,77,78,79,80,81,82,83,84,85,86,87]) involved in such processes.

In this study, we have used HUVEC as a model for the *in vivo* endothelium. It has been widely accepted to be a model system for studying vascular responses in diabetes and atherosclerosis [23]. The miRNA profiles between the different endothelial cell types are similar [26]. Nevertheless, there are differences between the different vasculature within the human circulatory system [4]. However, the scope of this paper was only to investigate and discuss a general response in the endothelium upon hyperglycemia. Thus, for identifying groups of miRNAs for vascular damage in specific target organs, further work must be performed on respective cell lines derived from the vessels native to the target organ of interest.

## 4. Materials and Methods

### 4.1. Cell Culture

Human Umbilical vein endothelial cells (HUVECs) were purchased from ATCC (CRL-1730) and grown in a T75 flask using 5 mM glucose Dulbecco’s Modified Eagle Medium (DMEM; Thermo Fisher Scientific, Waltham, MA, USA) supplemented with 2 mM l-glutamine, 10% fetal bovine serum (FBS) and 1% penicillin (100 IU/mL) and streptomycin (100 µg/mL) (Thermo Fisher Scientific) in the presence of 5% CO_2_ at 37 °C. Media were changed every 48 h until the cells reached 80%–90% confluence. The cells (between passages 3 to 6) were then sub-cultured in serum starved 5 mM glucose DMEM containing 1% FBS. After which, they were seeded at a density of 6 × 10^4^ cells/well in 24 well plates (Greiner bio-one, Cell star, Kremsmünster, Austria) and grown for another 24 h to reach 80%–90% confluence before treating them separately with media containing different concentrations (5, 10, 25 and 40 mM) of glucose for 6, 12, 24 and 48 h time intervals.

### 4.2. Glucose Uptake Measurement Assay

HUVECs were always grown first in 300 µL per well of DMEM containing 5 mM glucose and 1% FBS and then treated with different concentrations of glucose as above for 24 and 48 h. The total volume of media remained constant for all treatments. The media were then collected separately, cells washed twice with 1× PBS, then lysed with 1× lysis buffer and kept at −20 °C until needed. The amount of glucose taken up by the cells and that remaining in the medium were measured according to manufacturer’s protocol using glucose (HK) assay kit (GAHK-20; Sigma, Saint Louis, MO, USA) [128]. In brief, the collected cell lysates and media were diluted 1:1 ratio with sterile distilled water to measure the amount of glucose level as recommended by the manufacturer. In a fresh Eppendorf tube, 500 µL of glucose assay reagent was added along with 150 µL of a test sample, mixed well and incubated at room temperature for 15 min. The absorbance at a wavelength of 340 nm was then measured in a spectrophotometer (Model 680 Microplate Reader, Biorad, Hercules, CA, USA).

### 4.3. Quantification of Vascular Endothelial Growth Factor A (VEGFA) Release

VEGFA release in the cell culture media obtained after treatment of HUVECs with different concentrations of glucose was measured by an ELISA kit following manufacturer’s protocol (Invitrogen, Carlsbad, CA, USA) at 450 nm using a Microplate Reader (Model 680; Biorad, Hercules, CA, USA).

### 4.4. Cell Viability Assay

The cell viability was determined by 3-[4,5-dimethylthiazol-2-yl]-2,5-diphenyltetrazolium bromide (MTT; Sigma) uptake according to Armugam *et al.* [129]. HUVECs were seeded at a density of 6 × 10^4^ cells in 24 well plates and treated with different concentrations of glucose for 6, 12, 24 and 48 h. Ten microliters of MTT (10 mg/mL) was added one hour before the end point. The media were aspirated separately and the cells were lysed by adding 200 µL of Dimethyl Sulfoxide (DMSO; Sigma-Aldrich, St. Louis, MO, USA) to each well. The optical density of each sample was measured in a microplate reader (Model 680; Biorad) at 570 nm.

### 4.5. Cell Cytotoxicity Assay

Cell cytotoxicity was measured by the release of cytoplasmic enzyme, lactate dehydrogenase (LDH) into the cell culture medium. HUVECs incubated with Triton X-100, (Sigma-Aldrich, St. Louis, MO, USA) for 30 min at 37 °C was used as positive control. Fifty microliters from each culture medium was withdrawn and mixed with 50 µL of cytotoxicity detection assay kit reagent (Sigma-Aldrich, Roche Diagnostics, St. Louis, MO, USA) kept in a microtiter plate. The samples were mixed well and the absorption was measured at 490 nm in a microplate reader (Model 680; Biorad).

### 4.6. Total RNA Isolation

Total RNA (+miRNAs) was extracted from the cells by using Trizol Reagent (Invitrogen, Life Technologies, Carlsbad, CA, USA) according to the Jeyaseelan *et al.* [130]. The RNA concentration and purity were measured using Nanodrop ND-2000c spectrophotometry (Nanodrop Tech, Rockland, Del, Wilmington, DE, USA). The ratios of 260/280 were always kept within the range of 1.9–2.0 and RNA integrity was observed using denatured 1% agarose gel and 15% polyacrylamide gel electrophoresis.

### 4.7. miRNA Microarray Data and Statistical Analysis

LNA-modified oligonucleotide (Exiqon, Vedbaek, Denmark) probes for human miRNAs annotated in miRbase version 16.0 were used in the microarray that was carried out in our laboratory. A total RNA of 1 µg from three individual experiments (*n* = 3) were pooled for each concentration of glucose treatment (0–40 mM glucose) and their respective time intervals (6–48 h). The 3’end of RNA samples were labeled with Hy3 dye using miRCURY LNA power labeling kit (Exiqon). The labeled RNA was hybridized on miRCURY LNA arrays, using MAUI hybridization system (BioMicro Systems, Salt Lake City, UT, USA) for 17 h at 56 °C. The hybridized arrays were washed, fixed and scanned on InnoScan 700 microarray scanner (Innopsys, Carbonne, France). The digitalized images were captured and analyzed by MAPIX^®^4.5 (Innopsys) microarray image analysis software. Microarray analysis was carried out by background subtraction of the signal values, followed by One-way ANOVA analyses and hierarchical clustering [131]. Normalization was performed using an average of multiple endogenous controls. The hierarchical clustering method was used to detect the clustering pattern of samples across different concentrations of glucose treatment at various time intervals. The clustering was generated using TM4 MeV (Multiple Experimental Viewer) software (Dana-Farber Cancer Institute, Boston, MA, USA) [132] and statistical evaluations were performed using Microsoft Excel (2010) data analysis such as *t*-tests or in the case of multiple comparisons using One-way ANOVA with significance level *p* < 0.05. Differential expression analysis of the miRNAs was performed using the FDR (Benjamini-Hochberg False Discovery Rate) correction (*p* < 0.05) as in Partek^®^ Genomics Suite™ 6.6 Software (Partek Inc., St. Louis, MO, USA). *Post hoc* test was performed with Bonferroni correction to determine the difference between the different groups. Hierarchical clustering (HCL) and k-means clustering were performed using TIGR MeV (TMeV) software and Partek^®^ Genomics Suite™ 6.6 Software [133,134].

### 4.8. Biological Pathway Analysis (miRNA and mRNA)

DIANA (DNA Intelligent Analysis) miRPath [88] and miRWalk pathway analysis [89] were performed with the MicroT threshold cut-off value of 0.8 and *p*-value <0.05, for prediction of miRNA mediated pathway analysis. Similarly KEGG (Kyoto Encyclopedia of Genes and Genomes) pathway [90] was used for pathway analysis for mRNA and the top 20 pathways based on the enrichment scores were selected.

### 4.9. Assessment of Nuclear Morphology

To characterize the pattern of cell death, nuclear morphology was observed by Hoechst 33342 staining and fluorescence microscopy. HUVECs seeded and treated as for the microarray experiments were washed twice with 1x PBS and incubated with 0.1 µg/mL Hoechst 33342 (Biotium, Foster City, CA, USA) for 15 min in the dark and visualized under an Olympus IX51 microscope (Olympus, Shinjuku-ku, Tokyo, Japan) using DAPI (4′,6-diamidino-2-phenylindole) fluorescence filter. Digital Images were captured with 20× objective using Olympus DP71 digital camera and Olympus DP controller software program. Cells with the morphology of fragmented or condensed pyknotic nuclei were considered as apoptotic and counted using Image J software (Schneider, Madison, WI, USA) [135]. Each experiment was carried out in triplicates (*n* = 3).

### 4.10. Flow Cytometry

The confluent monolayer of HUVECs treated with different concentrations of glucose for 24 and 48 h after washing twice with 1× PBS were gently detached using 0.05% of trypsin. The cells were collected in 2 mL Eppendorf tubes and spun down at 800 rpm for 5 min. The cell pellets were suspended in 500 µL of 1× Annexin binding buffer and subjected to staining with 3 dyes: Annexin V, Ethidium Homodimer III, and DAPI, to detect apoptosis (Biotium) according to manufacturer’s protocol. Flow cytometric analysis was performed by analyzing 10,000 events on FACScan flow cytometer (BD biosciences, San Jose, CA, USA) and the data were processed and analyzed using summit 4.0 software package.

### 4.11. Caspase-3 Assay

To determine whether the cells undergo caspase dependent apoptosis upon glucose treatment, both the active form of caspase-3 and caspase-3 activity were measured in the total cell lysate using Invitrogen human active caspase-3 ELISA kit (KHO1091; Life technologies, Carlsbad, CA, USA) and (Alexis Corporation, Lausen, Switzerland), respectively, according to manufacturers’ protocol. Background fluorescence was measured in wells containing lysis buffer, assay buffer and the substrate without cell lysate and used for the normalization of the test samples. For active caspase-3 measurement readings at 450 nm were obtained using microplate spectrofluorometer (Spectra Gemini; Molecular devices, Sunnyvale, CA, USA). For caspase-3 activity, the fluorometric readings were measured at 405 nm absorption. All the measurements were carried out in triplicates and for 3 independent sets of experiments.

### 4.12. Real-Time Quantitative Polymerase Chain Reaction (qPCR)

Total RNA was isolated from cultured cells using TRIzol^®^ Reagent (Invitrogen, Life Technologies Corporation). Reverse transcription followed by real-time quantitative PCR (qRT-PCR) was carried out according to Jeyaseelan *et al.* [130]. Gene specific primers designed using PrimerExpress software (Version 3.0) from Applied Biosystems (Carlsbad, CA, USA) have been used for qRT-PCR on an Applied Biosystems 7900 sequence detection system (Applied Biosystems). The miRNA microarray results were validated with stem-loop real time qPCR. Ten nanograms of total RNA was reverse transcribed and used for stem loop PCR. GAPDH mRNA was used as the endogenous control for both miRNA and mRNA measurements. Each reaction was performed in triplicates (*n* = 3). Statistical significance analysis between the control (treated with 5 mM glucose) *vs.* test (10, 25 or 40 mM glucose) was carried out using student *t*-test.

## 5. Conclusions

In summary, our study describes the miRNA dysregulation in hyperglycemic conditions induce endothelial dysfunction and apoptosis while highlighting that these miRNAs could also function as “glucose responsive miRNAs”. Furthermore, these miRNAs being detectable (in blood) in the pre-diabetic condition (IFG) indicates their possible role as potential biomarkers in the early diagnosis of diabetes.

## Figures and Tables

**Table 1:** Ten miRNAs selected from miRNA microarray analyses (human, rat and HUVECs) with statistical significance; *p*-value < 0.05.

miRNAs	Human IFG	Human T2DM	Rat T2DM Model	HUVECs			
				25 mM Glucose		40 mM Glucose	
				24 h	48 h	24 h	48 h
miR-26a-5p	−1.64	1.80	1.40	1.27	1.55	1.84	2.12
miR-26b-5p	2.37	1.52	1.33	1.23	1.42	1.44	2.24
miR-29b-3p	1.45	1.96	2.02	1.69	2.19	2.42	2.86
miR-29c-3p	1.55	2.35	2.45	1.86	2.21	2.65	3.25
miR-125b-1-3p	−1.55	1.70	2.23	1.27	1.97	1.41	4.30
miR-130b-3p	1.87	1.89	1.54	1.11	1.22	1.27	1.57
miR-140-5p	1.62	1.69	1.71	1.53	1.92	2.27	2.68
miR-192-5p	1.34	2.25	1.87	1.45	1.87	1.97	2.41
miR-221-3p	1.74	2.01	2.16	1.44	1.95	2.65	3.10
miR-320a	−1.58	1.81	2.87	1.59	1.90	1.81	2.46

## Supplementary Materials

Supplementary materials can be found at http://www.mdpi.com/1422-0067/17/4/518/s1.

## Abbreviations

T2DM	Type 2 Diabetes Mellitus
IFG	Impaired Fasting Glucose
ECs	Endothelial Cells
MicroRNAs	miRNAs
GEO	Gene Expression Omnibus
DEVD-AMC	*N*-Acetyl-Asp-Glu-Val-Asp-7-amido-4-Methylcoumarin
Ac-DEVD-CHO	Acetyl-Asp-Glu-Val-Asp-1-aldehyde
HK	Hexokinase
