# Supplementary material for: MicroRNAs in Hyperglycemia Induced Endothelial Cell Dysfunction
Source: Int J Mol Sci. 2016 Apr 7;17(4):518. doi: 10.3390/ijms17040518 (PMC4848974; doi:10.3390/ijms17040518)
Supplement: Supplementary file 1 [file ijms-17-00518-s001.pdf]

Supplementary Materials: MicroRNAs in Hyperglycemia Induced Endothelial Cell Dysfunction

Maskomani Silambarasan, Jun Rong Tan, Dwi Setyowati Karolina, Arunmozhiarasi Armugam, Charanjit Kaur and Kandiah Jeyaseelan

Table S1. miRNA microarray results from HUVECs cells subjected to different hyperglycemic conditions and treatment intervals. Fold change values with statistical significance (p < 0.05) in expression are shown below. The values were normalized against control where the cells were treated with 5 mM glucose for different time intervals.

	hsa-miRNA	5 mM	10 mM	25 mM	40 mM
	Glucose treatment for 6 h				
1	miR-1260a	1.00	1.49	1.95	2.33
2	miR-1270	1.00	2.03	2.42	3.80
3	miR-20b-5p #	1.00	1.96	2.05	2.23
4	miR-376a-3p	1.00	1.80	1.91	2.11
5	miR-570-3p #	1.00	1.19	3.40	5.48
6	miR-616-3p	1.00	1.20	1.69	2.91
	Glucose treatment for 12h				
7	miR-1267	1.00	1.38	2.39	2.59
8	miR-1284	1.00	1.32	1.35	1.56
9	miR-1304-5p	1.00	1.41	1.54	1.60
10	miR-133b #	1.00	1.12	1.24	1.43
11	miR-148a-5p #	1.00	1.06	1.57	1.66
12	miR-148b-3p	1.00	1.07	1.19	1.36
13	miR-183-3p	1.00	1.07	1.23	1.28
14	miR-198	1.00	1.33	1.77	1.93
15	miR-203a	1.00	1.13	1.88	1.54
16	miR-22-5p	1.00	1.26	2.05	2.09
17	miR-221-5p	1.00	1.20	1.60	1.63
18	miR-23a-5p	1.00	1.28	2.11	2.24
19	miR-29b-1-5p	1.00	1.43	1.58	1.61
20	miR-320a #	1.00	1.27	1.44	1.76
21	miR-320b	1.00	1.13	1.22	1.39
22	miR-320c	1.00	1.15	1.38	1.43

	hsa-miRNA	5 mM	10 mM	25 mM	40 mM
38	miR-192-5p #	1.00	1.26	1.45	1.97
39	miR-20b-5p #	1.00	1.56	1.68	1.81
40	miR-219a-1-3p	1.00	1.87	1.88	2.81
41	miR-221-3p #	1.00	1.11	1.27	1.41
42	miR-222-3p	1.00	1.06	1.11	1.52
43	miR-26a-5 p#	1.00	1.13	1.27	1.84
44	miR-26b-5p #	1.00	1.10	1.23	1.44
45	miR-29b-3p #	1.00	1.25	1.69	2.42
46	miR-29c-3p #	1.00	1.26	1.86	2.65
47	miR-320a #	1.00	1.38	1.59	1.81
48	miR-34c-5p	1.00	1.85	2.29	4.60
49	miR-376b-3p	1.00	1.29	1.44	1.73
50	miR-502-5p	1.00	1.12	1.39	2.10
51	miR-518a-5p/ miR-527	1.00	1.08	1.35	2.33
52	miR-519e-3p	1.00	1.13	1.42	2.23
53	miR-570-3p #	1.00	1.76	2.33	6.40
54	miR-573	1.00	2.11	2.40	6.03
55	miR-611	1.00	1.69	1.89	3.50
56	miR-645	1.00	1.94	2.55	2.54
	Glucose treatment for 48h				
57	miR-125b-1-3p #	1.00	1.12	1.22	1.57
58	miR-130b-3p #	1.00	1.58	1.92	2.68
59	miR-133a	1.00	1.14	1.38	1.81
60	miR-133b #	1.00	1.01	1.13	1.70

23	miR-574-3p	1.00	1.42	1.59	1.78	61	miR-140-5p #	1.00	1.79	1.95	3.10
Glucose treatment for 24h						62	miR-15b-5p	1.00	1.11	1.32	1.74
24	miR-1183	1.00	1.25	2.17	3.88	63	miR-17-3p	1.00	1.11	1.28	1.79
25	miR-1227-3p	1.00	1.62	1.80	2.98	64	miR-181a-5p	1.00	1.12	1.42	1.81
26	miR-1252-3p	1.00	1.91	2.10	2.85	65	miR-181b-5p	1.00	1.10	1.50	1.66
27	miR-125b-1-3p #	1.00	1.04	1.11	1.27	66	miR-18b-5p	1.00	1.26	1.38	1.74
28	miR-1262	1.00	1.40	1.65	2.63	67	miR-192-5p #	1.00	1.21	1.87	2.41
29	miR-1266-5p	1.00	1.07	2.70	4.72	68	miR-19a-3p	1.00	1.16	1.26	1.43
30	miR-130b-3p #	1.00	1.26	1.53	2.27	69	miR-19b-3p	1.00	1.21	1.22	1.59
31	miR-132-5p	1.00	1.26	1.61	2.40	70	miR-21-5p	1.00	1.08	1.13	1.93
32	miR-133a-3p	1.00	1.34	1.39	1.76	71	miR-221-3p #	1.00	1.38	1.97	4.30
33	miR-140-5p #	1.00	1.28	1.44	2.65	72	miR-26a-5p #	1.00	1.30	1.55	2.12
34	miR-148a-5p #	1.00	1.36	1.44	1.89	73	miR-26b-5p #	1.00	1.22	1.42	2.24
35	miR-181a-3p	1.00	1.07	1.12	1.51	74	miR-29b-3p #	1.00	1.35	2.19	2.86
36	miR-181a-2-3p	1.00	1.31	1.40	2.50	75	miR-29c-3p #	1.00	1.44	2.21	3.25
37	miR-187-5p	1.00	1.17	1.20	1.81	76	miR-320a #	1.00	1.40	1.90	2.46

Of the 76 miRNAs listed, 62 miRNAs have been found to be differentially expressed in more than one time point. These miRNAs have been indicated with #.

Table S2. The selected 10 miRNAs and their expression values as well as their validated target mRNAs and functions. The expression levels of miRNAs upon different glucose treatments are shown as fold changes normalized against the control (5 mM glucose treatment).

miRNAs	Validated Targets	Gene Description	Gene Function	40 mM Glucose				25 mM Glucose				10 mM Glucose			
				6 h	12 h	24 h	48 h	6 h	12 h	24 h	48 h	6 h	12 h	24 h	48 h
	HMGA2	high mobility group AT-hook 2 [38]	Down-regulation of HMGA2 promotes endothelial progenitor cell senescence [39]												
	PTEN	phosphatase and tensin homolog [40]	Endothelial specific deletion of PTEN prompts angiogenesis [41] and tumor growth [42]												
miR-26a-5p	RB1	retinoblastoma 1 [40]	RB1 protects endothelial cell from apoptosis [43] and senescence [44]	1.02	1.29	1.84	2.12	1.06	1.06	1.27	1.55	1.00	1.17	1.13	1.29
	EZH2	enhancer of zeste 2 polycomb repressive complex 2 subunit [45]	Decrease in EZH2 promotes endothelial cell to quiescence state [46] Gestational diabetes impairs HUVECs function via inhibition of EZH2 [47]												
	MCL1	myeloid cell leukemia 1 [48]	High expression of MCL1 induces tumor progression and angiogenesis [49]												
	EphA2	EPH receptor A2 [50]	Increased expression of EphA2 mediates vascular leakage [51]												
miR-26b-5p	PTEN	phosphatase and tensin homolog [52]	Endothelial specific deletion of PTEN prompts angiogenesis [41] and tumor growth [42]	1.05	1.36	1.44	2.24	1.03	−1.06	1.23	1.42	1.06	1.08	1.10	1.21
	RB1	retinoblastoma 1 [53]	RB1 protects endothelial cell from apoptosis [43] and												

			senescence [44]												
	EZH2	enhancer of zeste 2 polycomb repressive complex 2 subunit [54]	Decrease in EZH2 promotes endothelial cell to quiescence state [95 46], Gestational diabetes impairs HUVECs function via inhibition of EZH2 [47]												
	MCL1	myeloid cell leukemia 1 [55]	High expression of MCL1 induces tumor progression and angiogenesis [49]												
miR-29b-3p	MCL1	myeloid cell leukemia 1 [56]	High expression of MCL1 induces tumor progression and angiogenesis [49]	1.08	1.20	2.42	2.86	1.15	1.61	1.69	2.19	1.06	1.52	1.25	1.35
	BCL2	B-cell lymphoma 2 [57]	Decreases in BCL2 induces endothelial apoptosis [58]												
	HDAC4	histone deacetylase 4 [59]	HDAC4 represses VEGFA expression by modulates RUNX2 activity [60]												
	VEGFA	vascular endothelial growth factor A [61]	VEGFA induces pathological angiogenesis [62]												
	PTEN	phosphatase and tensin homolog [63]	Endothelial specific deletion of PTEN prompts angiogenesis [41] and tumor growth [42]												
miR-29c-3p	MCL1	myeloid cell leukemia 1 [64]	High expression of MCL1 induces tumor progression and angiogenesis [49]	1.06	1.21	2.65	3.25	1.01	1.34	1.86	2.21	1.09	1.45	1.26	1.44
	BCL2	B-cell lymphoma 2 [64]	Decreases in BCL2 induces endothelial apoptosis [58]												
	HDAC4	histone deacetylase 4 [65]	HDAC4 represses VEGFA expression by modulates RUNX2 activity [60]												
	PTEN	phosphatase and tensin homolog [66]	Endothelial specific deletion of PTEN prompts angiogenesis [41] and tumor growth [42]												
miR-125b-1-3p	S1PR1	sphingosine-1-phosphate receptor 1 [67]	Hyperglycemia induced decrease in S1PR1 causes endothelial dysfunction [68]	1.07	1.13	1.41	4.30	1.09	−1.09	1.27	1.97	1.13	−1.08	1.04	1.12
miR-130b-3p	PTEN	phosphatase and tensin homolog [69]	Endothelial specific deletion of PTEN accelerates angiogenesis [41] and tumor growth [42]	1.03	1.12	1.27	1.57	1.08	1.22	1.11	1.22	1.17	1.10	1.26	1.58
	STAT3	signal transducer and activator of transcription 3 [70]	Interleukin 17 induces endothelial STAT3 mediated endothelial activation [71] and pathological angiogenesis [72]												
	PPARG	peroxisome proliferator-activated receptor gamma [73]	PPARG protects blood brain barrier breakdown [74], Disruption of endothelial PPARG induces atherogenesis [75]												
miR-140-5p	VEGFA	vascular endothelial growth factor A [61]	VEGFA induces pathological angiogenesis [62]	1.03	1.80	2.27	2.68	1.09	2.15	1.53	1.92	1.09	1.06	1.28	1.79
	HDAC4	histone deacetylase 4 [76]	HDAC4 represses VEGFA expression by modulates RUNX2 activity [60]												
miR-192-5p	BCL2	B-cell lymphoma 2 [77]	Decreases in BCL2 induces endothelial apoptosis [58]	1.03	−1.06	1.97	2.41	1.17	1.24	1.45	1.87	1.01	−1.07	1.26	1.21
	RB1	retinoblastoma 1 [78]	RB1 protects endothelial cell from apoptosis [43] and senescence [44]												
miR-221-3p	PTEN	phosphatase and tensin homolog [79]	Endothelial specific deletion of PTEN accelerates angiogenesis [41] and tumor growth [42]	1.09	−1.09	2.65	3.10	1.16	-1.13	1.44	1.95	1.09	1.21	1.11	1.38
	RB1	retinoblastoma 1 [80]	RB1 protects endothelial cell from apoptosis [43] and senescence [44]												
	PAK1	p21 protein (Cdc42/Rac)-activated	PAK1 enhances endothelial barrier function by modulates cortical actin structure [82,83]												

		kinase 1 [81]													
miR-320a	NRP1	neuropilin 1 [84]	NRP1 promotes endothelial tip cell function during angiogenesis [85], NRP1 acts as the receptor for VEGFA and induces angiogenesis [86]	1.01	1.75	1.81	2.46	1.13	1.44	1.59	1.90	1.05	1.27	1.38	1.54
	MCL1	myeloid cell leukemia 1 [87]	High expression of MCL1 induces tumor progression and angiogenesis [49]												

Table S3. *In silico* pathway prediction—Independent pathway analysis for miRNAs and mRNAs that are dysregulated in both human IFG/T2DM and rat T2DM model.

Commonly Upregulated 25 miRNAs in Human T2DM Subjects and Rat T2DM Model				
	**KEGG Pathway**	**miRNAs**	**Genes**	***p*-Value**
1	Regulation of actin cytoskeleton	25	109	1.30×10^{-33}
2	Focal adhesion	25	99	4.38×10^{-30}
3	Endocytosis	25	93	8.28×10^{-16}
4	Axon guidance	25	72	6.01×10^{-29}
5	Pathways in cancer	24	165	4.72×10^{-39}
6	P13K-Akt signaling pathway	24	154	1.42×10^{-29}
7	MAPK signaling pathway	24	118	7.23×10^{-19}
8	Wnt signaling pathway	24	81	3.05×10^{-27}
9	Ubiquitin mediated proteolysis	24	68	6.33×10^{-25}
10	Neurotrophin signaling pathway	24	65	2.98×10^{-26}
11	TGF-β signaling pathway	24	55	2.26×10^{-24}
12	Apoptosis	23	53	2.00×10^{-21}
13	Chronic myeloid leukemia	23	43	6.76×10^{-18}
14	Renal cell carcinoma	23	40	5.64×10^{-17}
15	Long term potentiation	23	40	8.28×10^{-16}
16	Melanoma	23	38	8.28×10^{-16}
17	Gap junction	21	45	1.36×10^{-14}
18	Gastric acid secretion	21	39	1.24×10^{-15}
19	mTOR signaling pathway	21	37	1.13×10^{-14}
20	Colorectal cancer	21	35	2.96×10^{-15}

Commonly Downregulated 27 miRNAs in Human T2DM Subjects and Rat T2DM Model				
	**KEGG Pathway**	**miRNAs**	**Genes**	***p*-Value**
1	MAPK signaling pathway	24	130	1.85×10^{-30}
2	Neurotrophin signaling pathway	24	71	1.41×10^{-29}
3	Wnt signaling pathway	23	84	6.72×10^{-37}
4	Apoptosis	23	50	6.24×10^{-21}
5	P13K-Akt signaling pathway	22	169	4.80×10^{-64}
6	Pathways in cancer	22	166	4.18×10^{-42}
7	Focal adhesion	22	102	2.27×10^{-41}
8	Regulation of actin cytoskeleton	22	101	1.37×10^{-20}
9	Ubiquitin mediated proteolysis	22	68	4.95×10^{-21}
10	Dopaminergic synapse	22	67	1.53×10^{-22}
11	Prostate cancer	22	55	4.55×10^{-23}
12	Gap junction	22	47	1.05×10^{-21}
13	Phosphatidylinositol signaling system	22	47	2.74×10^{-20}
14	Insulin signaling system	21	70	1.30×10^{-28}
15	Small cell lung cancer	21	49	1.89×10^{-20}
16	ErbB signaling pathway	21	48	5.49×10^{-21}
17	TGF-beta signaling pathway	21	47	2.49×10^{-21}
18	Chronic myeloid leukemia	21	43	2.05×10^{-19}
19	Renal cell carcinoma	21	41	7.10×10^{-20}
20	p53 signaling	19	44	4.39×10^{-19}

Top 20 Commonly Dysregulated mRNA Pathways in Human IFG and T2DM Subjects			
	**KEGG Pathway**	**Genes**	***p*-Value**
1	Metabolic pathways	868	5.67×10^{-236}
2	PI3K-Akt signaling pathway	283	3.06×10^{-100}
3	MAPK signaling pathway	267	2.66×10^{-65}

4	Insulin signaling pathway	260	4.09×10^{-94}
5	Apoptosis	259	4.36×10^{-89}
6	Chemokine signaling pathway	254	4.45×10^{-86}
7	Focal adhesion	192	7.13×10^{-68}
8	Regulation of actin cytoskeleton	199	2.16×10^{-64}
9	Oxidative phosphorylation	189	1.83×10^{-62}
10	ErbB signaling pathway	180	4.58×10^{-62}
11	Calcium signaling pathway	171	6.05×10^{-62}
12	Adherens junction	163	6.71×10^{-46}
13	VEGF signaling pathway	151	1.04×10^{-48}
14	Type II diabetes mellitus	153	4.35×10^{-60}
15	Wnt-signaling pathway	147	3.91×10^{-56}
16	TGF-beta signaling pathway	146	8.91×10^{-51}
17	Neurotrophin signaling pathway	130	1.13×10^{-46}
18	mTOR signaling pathway	121	3.85×10^{-42}
19	Long-term potentiation	127	1.71×10^{-44}
20	Tight junction	83	2.01×10^{-29}

Table S4. Human mRNA microarray (Adapted from Karolina *et al.*; Reference [20] in the main text) mRNAs with background subtracted mean signal intensities ≥300 are included. Some of the mRNAs dysregulated in IFG and T2DM patients are shown. Data are expressed as fold changes.

mRNA	1 IFG	2 IFG	3 IFG	4 IFG	5 IFG	1 T2D	2 T2D	3 T2D	4 T2D	5 T2D	6 T2D	7 T2D	8 T2D
NOS3	−3.01	−3.06	−3.11	−3.00	−3.12	−3.06	−3.02	−3.14	−3.09	−3.03	−3.21	−3.15	−3.07
EDN1	3.26	3.34	3.35	3.22	3.28	3.37	3.33	3.07	3.03	3.16	3.11	3.18	3.20
ERG	−3.08	−3.25	−3.28	−3.18	−3.28	−3.27	−3.16	−3.31	−3.33	−3.35	−3.33	−3.32	−3.11
VEGFA	1.50	1.27	1.66	1.48	1.64	1.44	1.51	1.43	1.28	1.6	1.34	1.33	1.33
RHOA	52.20	41.97	56.34	39.46	69.9	41.77	47.4	43.06	43.67	46.74	39.65	47.51	45.20
ROCK2	377.73	312.76	521.40	297.16	513.38	442.63	519.69	254.48	331.87	277.6	276.42	304.67	387.21
CCL5	210.81	189.76	175.84	184.57	209.19	137.59	230.71	148.52	140.59	191.3	150.21	197.4	160.80
TLR4	4.01	2.95	4.61	3.09	4.82	2.14	3.21	3.83	3.13	4.82	3.52	3.91	2.50
NFKB1	42.69	31.34	43.28	27.30	46.26	30.14	32.8	33.43	34.54	34.01	32.93	32.15	32.78
IL1B	22.58	11.07	13.26	8.59	17.30	8.12	9.05	15.00	14.80	14.32	12.34	14.89	11.71
BNIP3	2.72	2.27	2.99	2.09	2.90	2.92	3.75	1.93	1.63	3.61	2.29	2.07	2.58
DNM1L	2.32	1.70	2.55	1.88	2.46	2.05	3.01	1.79	1.90	2.76	2.38	1.78	2.06
BCL2	−5.64	−4.31	−5.42	−4.46	−4.62	−7.05	−6.94	−7.02	−7.42	−8.60	−8.49	−8.16	−8.43
MCL1	−8.31	−8.39	−8.13	−8.38	−8.89	−10.92	−10.89	−10.35	−10.26	−10.4	−10.39	−10.59	−10.55
CASP3	9.37	7.29	8.97	8.23	7.85	9.07	8.93	8.7	6.81	14.47	7.18	8.53	5.88

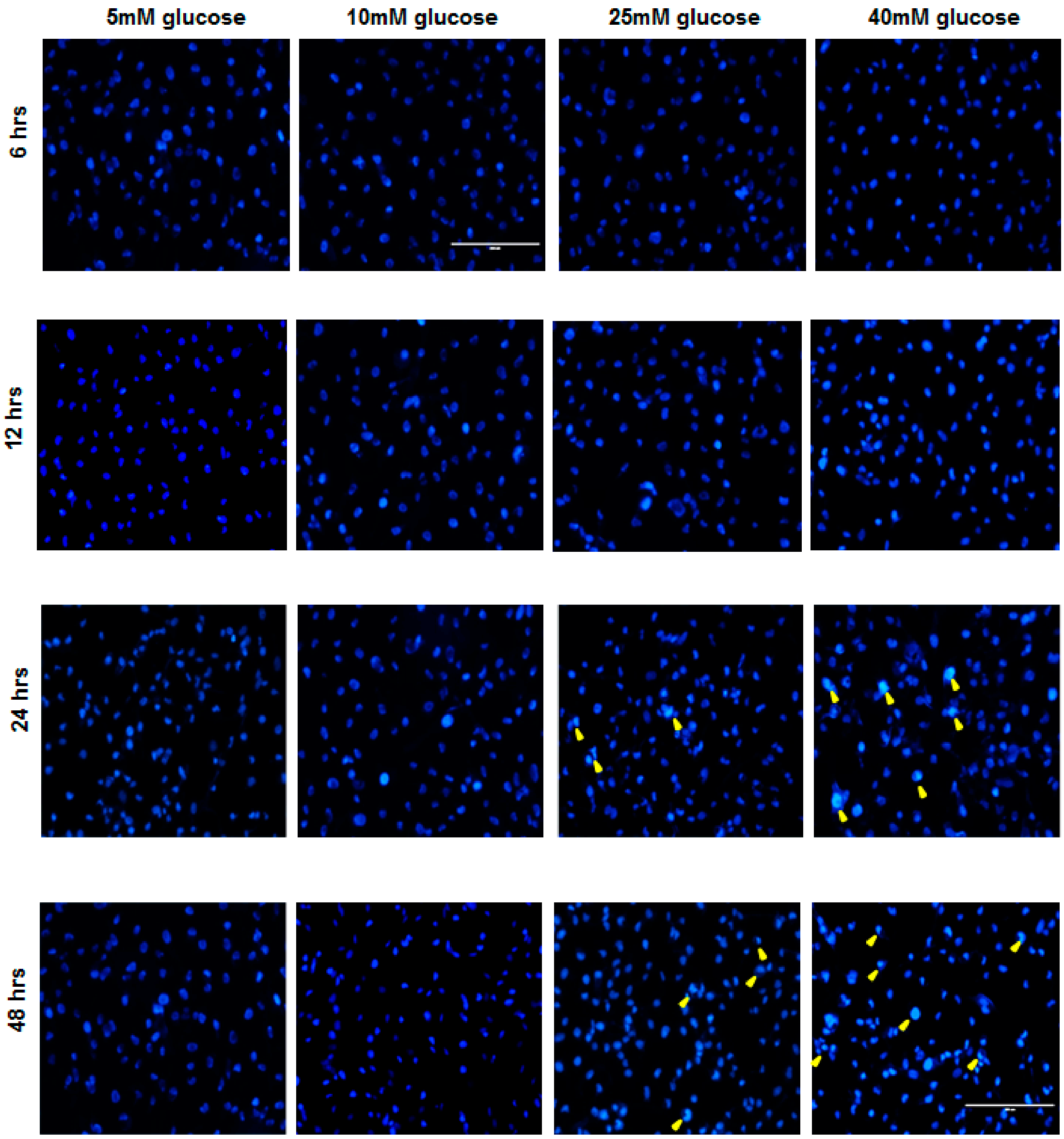

Figure S1. Fluorescence microscopy for DAPI nuclear staining. Cells were subjected to different glucose treatments (5–40 mM) and collected at various time-points (6–48 h). The images were captured using Olympus IX51 microscope (objective 20× magnification). Healthy nuclei can be observed for 5 mM glucose treatments (6, 12, 24 and 48 h). A significant amount of shrunken/condensed and pyknotic nuclei can be seen only under hyperglycemic conditions (24 and 48 h).

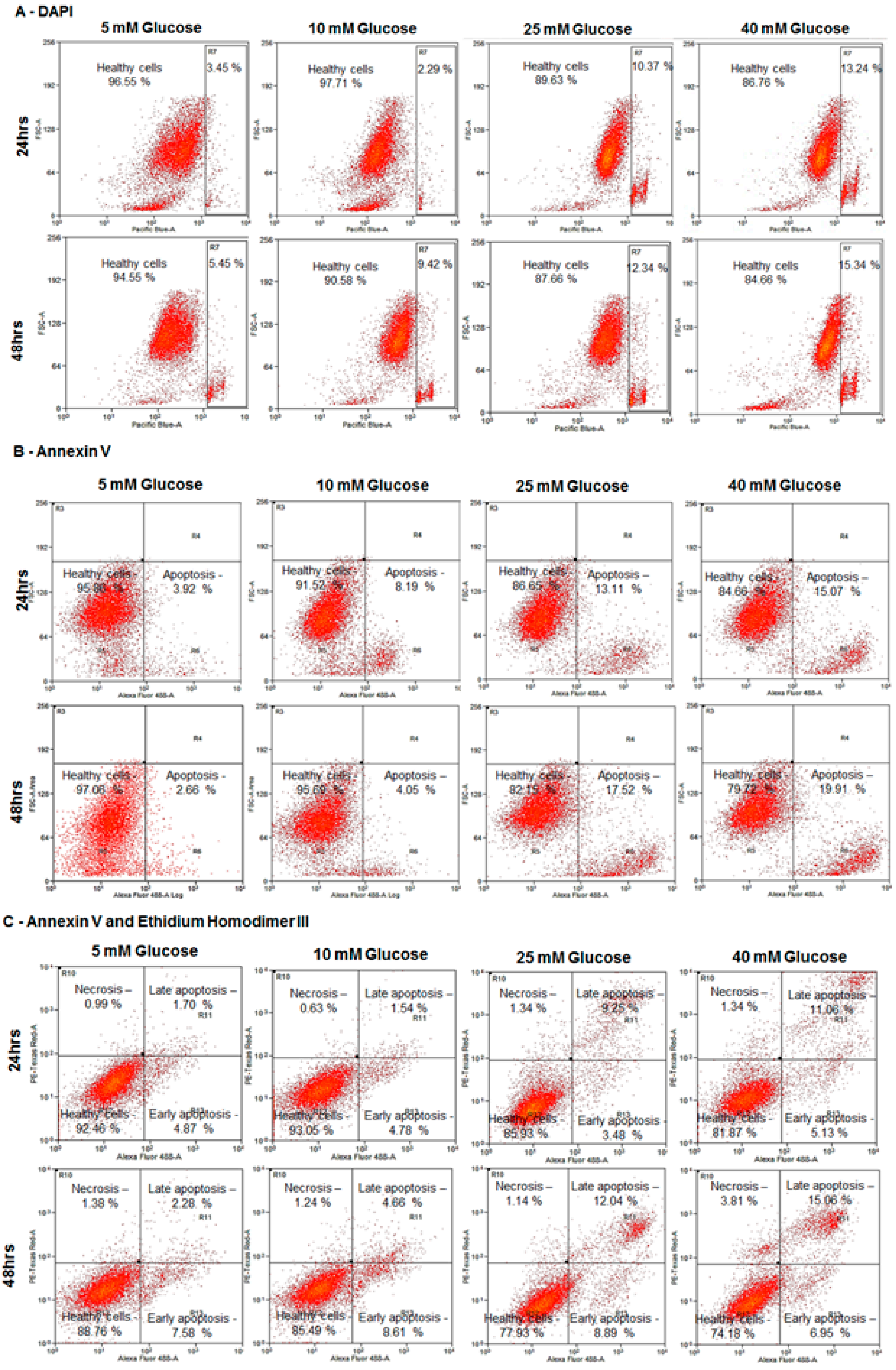

Figure S2. Fluorescence activated cell sorting (FACS). Cells were subjected to different glucose treatments (5–40 mM) and collected at various time-points (6–48 h). Dot-plot graph on FACS for cells stained with (**A**) DAPI (**B**) Annexin V (**C**) Annexin V and Ethidium Homodimer staining. The percentages of healthy and apoptotic cells are indicated in their respective quadrants.

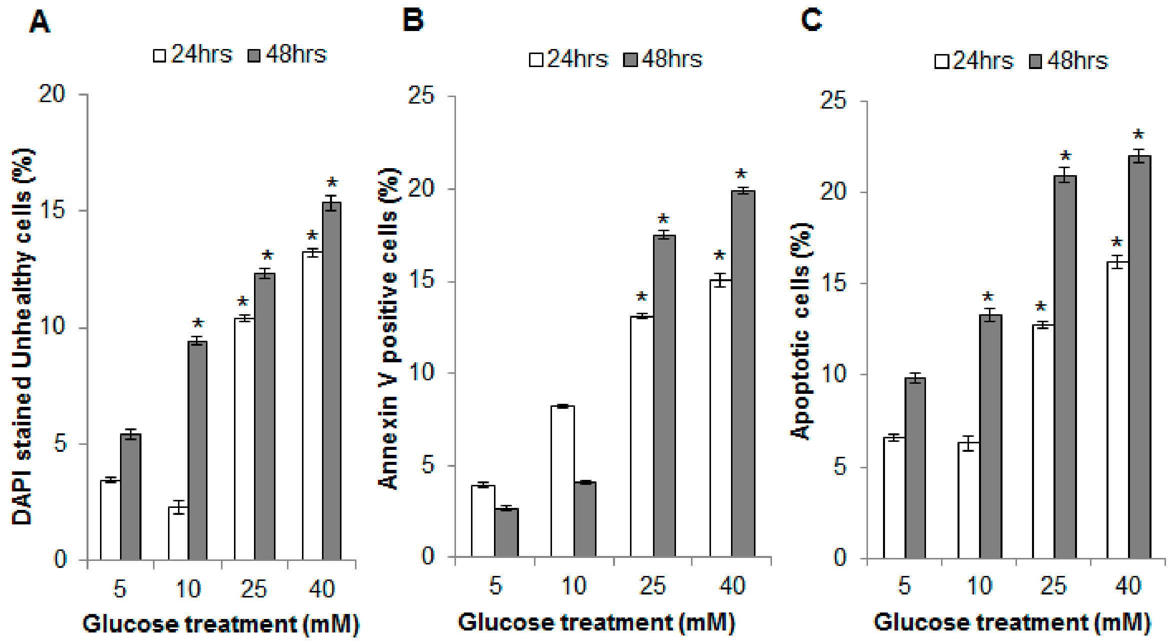

Fluorescence Associated Cell Sorting (FACS) – Statistical analysis												
	DAPI stained Unhealthy cells (%)		*t-test (p-value)*		Annexin V positive cells (%)		*t-test (p-value)*		Annexin V and Ethidium Homo dimer III positive cells (%)		*t-test (p-value)*	
Treatment	24hrs	48hrs	24hrs	48hrs	24hrs	48hrs	24hrs	48hrs	24hrs	48hrs	24hrs	48hrs
5mM glucose	3.45	5.45	*NA*	*NA*	3.92	2.66	*NA*	*NA*	6.57	9.86	*NA*	*NA*
10mM glucose	2.29	9.42	*0.056*	*0.000*	8.19	4.05	*0.000*	*0.000*	6.32	13.27	*0.002*	*0.000*
25mM glucose	10.37	12.34	*0.000*	*0.000*	13.11	17.52	*0.000*	*0.000*	12.73	20.93	*0.000*	*0.000*
40mM glucose	13.24	15.34	*0.000*	*0.000*	15.07	19.91	*0.000*	*0.000*	16.19	22.01	*0.000*	*0.000*

Figure S3. Statistical analysis of FACS study: The percentage of apoptosis at 25 and 40 mM glucose treatments are statistically significant compared to 5mM glucose control. (**A**) Percentage of DAPI stained cells unhealthy (R7 quadrant, Figure S2A); (**B**) Percentage of Annexin V positive cells (R6 quadrant, Figure S2B); (**C**) Percentage of Annexin V positive and Ethidium Homo dimer III stained cells (R11 and R13 quadrants, Figure S2C). Data presented as mean ± SEM (n = 3). *: Indicates statistical significance, $p < 0.05$.

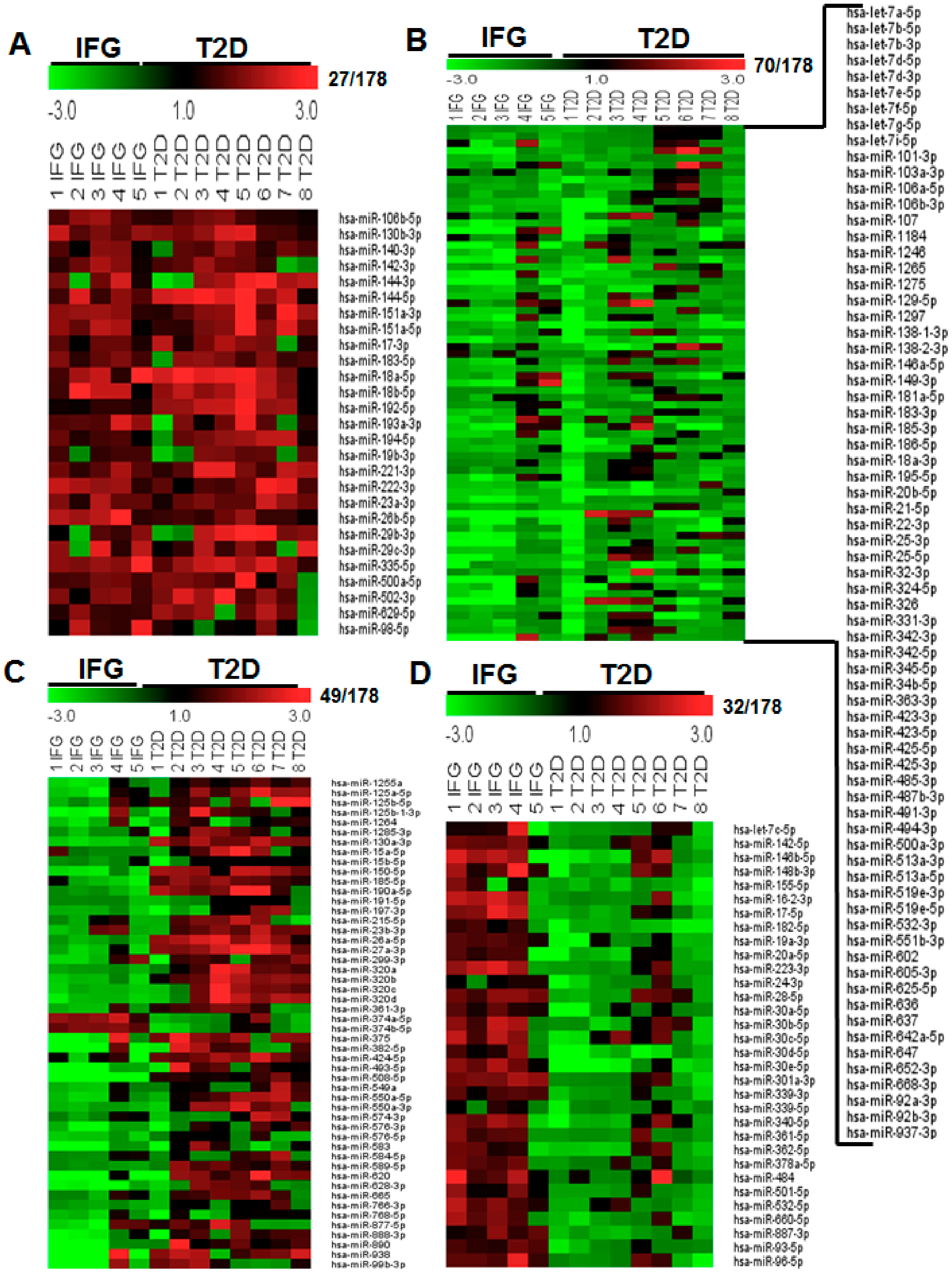

Figure S4. Categorization of 177 human microRNA expression profile based on IFG and T2DM. miRNAs with background subtracted mean signal intensities ≥300 are included. Heat map of selected miRNAs dysregulated in human T2DM. miRNAs that showed differential expression are grouped into 4 categories (**A**–**D**). miRNAs that remained: (**A**) upregulated in both IFG and T2DM against controls; (**B**) downregulated in both IFG and T2DM; (**C**) downregulated in IFG but upregulated in T2DM; (**D**) upregulated in IFG but downregulated in T2DM. Data are expressed as fold change. Red represents up-regulation; green indicates down-regulation and grey—not detected.
